# Comment on “Human‐directed attachment behaviour in wolves suggests standing ancestral variation for human‐dog attachment bonds”

**DOI:** 10.1002/ece3.10514

**Published:** 2023-09-19

**Authors:** Márta Gácsi, Ádám Miklósi, József Topál

**Affiliations:** ^1^ ELKH‐ELTE Comparative Ethology Research Group Budapest Hungary; ^2^ Department of Ethology Eötvös Loránd University Budapest Hungary; ^3^ Institute of Cognitive Neuroscience and Psychology, Research Centre for Natural Sciences Budapest Hungary

**Keywords:** Zoology

## Abstract

In their recent paper, Hansen Wheat et al. (*Ecology and Evolution*, 2022, **12**, e9299) claimed that hand raised 23‐week‐old wolves showed the same attachment behaviour towards their handler in the Strange Situation Test (SST) (*Determinants of infant behavior*, 1969, **4**, 111) as dogs. At first glance, their results seem to contradict previous findings that domestication caused a unique change in social‐affiliative behaviours in dogs (*Animal Behaviour*, 2005, **70**, 1367). We argue that no persuading evidence was presented to claim that “wolves can show attachment behaviours towards humans comparable to those of dogs”. When dealing with a behaviour system (*Child Development*, 1977, **48**, 1184), the subjects' behaviour must meet consistent criteria (*Behavioural and Brain Science*, 1978, **3**, 417), and a few behavioural preferences should not be used to claim the presence of an attachment system, especially, if the experiment violates basic assumptions of the original test. We believe the intriguing scientific question is whether the dog‐owner relationship is qualitatively different from what could be observed in the wolf‐hand raiser relation. Assessing all available data, our answer is still yes; dogs are unique in this respect.

In their recent paper, Hansen Wheat et al. (2022) claimed that hand raised 23‐week‐old wolves showed the same attachment behaviour towards their handler in the Strange Situation Test (SST) (Ainsworth & Wittig, 1969) as dogs. At first glance, their results seem to contradict previous findings that domestication caused a unique change in social‐affiliative behaviours in dogs (Topál et al., 2005). They suggest that “wolves can show attachment behaviours towards humans comparable to those of dogs … refuting claims that this phenotype evolved after dog domestication was initiated”. The purpose of our commentary is not to criticise the findings presented in Hansen Wheat et al., but to highlight weaknesses in their interpretation and conclusions, which give the false impression that their data provide solid scientific evidence for “dog‐like” attachment behaviour towards humans in wolves.

In the original formulation, Bowlby (1969) described *attachment* as a behaviour system, whose main function is to control the infant‐parent distance. At least in humans, the attachment behaviour system also regulates the adult‐adult relationship, for example, in romantic couples (Simpson, 2017). Thus, rather than saying that a species possesses, does not possess or has a “rudimentary” attachment behaviour system, it is more precise to define (if possible) which kind of features are functional.

Conceptually, the authors refer to some “*collective evidence”* from previous dog‐wolf comparative studies; however, neither the results nor the interpretations of these studies are properly reflected in their arguments. The authors claim that “growing collection of evidence that wolves are capable of expressing attachment behaviour towards human‐caregivers (Hall et al., 2015; Lenkei et al., 2020; Ujfalussy et al., 2017)” highlight that “wolves across a wide range of ontogenetic stages, and not just very young wolf puppies, possess this capability”. In fact, the Hall et al. (2015) study was conducted on 3–7‐week‐old wolf pups, and though they showed what was called “attachment‐like” behaviours (preferential greeting of the caregiver and more proximity/contact seeking with them after separation), this type of attachment towards the caregiver is common in mammalian offspring during the pre‐weaning period. The Lenkei et al. (2020) study claimed that in their experiment wolves showed the “capacity to form at least some features of attachment with humans”. Indeed, wolves responded differently to being separated from an unfamiliar person versus from the handler, while the other person was holding the leash. However, it would have been important to get a somewhat more complex picture of the subjects' behaviour in this situation, for example, after a while allowing the wolves to follow the person who left (contact seeking) or go elsewhere (associating the caregiver with the opportunity to walk). The Ujfalussy et al. (2017) study was unequivocally not set to observe wolves' attachment behaviour at all, but focused solely on their greeting behaviour towards differently related people in their familiar environment. Even the authors did not interpret their results in the framework of attachment; they reported *preference* for the hand‐raiser in this situation. In summary, we fully agree that it is important to assess human‐wolf (or cat, or horse) bonding, but if non‐standard methods are used, the observed behaviour patterns or questionnaire responses should be interpreted more cautiously without using the term *attachment* (see e.g., Burkhard et al., 2023; Ujfalussy et al., 2017).

Addressing the findings of the Hansen Wheat et al. study, we argue that the rather limited validity of the results strongly questions the claims on similar attachment in dogs and wolves, thus their interpretation of the data in an evolutionary framework is premature. Basically, the findings cannot be compared with previous results of SSTs performed on dogs/wolves due to the important conceptual/methodological flaws of the study. In contrast to the authors' claims, the Hansen Wheat et al. study is not a replication of the adapted SST version, carried out by Topál et al. (2005) to compare young hand‐raised wolves and dogs, because of the fundamental methodological differences that violate the basic criteria of the SST paradigm. These assumptions are the (i) use of an *unfamiliar* place (because separation from the attachment figure at the unfamiliar place is supposed to induce “*moderate stress*”, which is essential to activate the attachment behaviour) (ii) interaction with a stranger vs. a *dedicated caretaker* (*the* hand‐raiser/owner).

In the Hansen Wheat study, the SST was carried out at a place that was *familiar* for all subjects, even though in all studies investigating attachment in infants or dogs the test was conducted at an *unfamiliar* test location. The unfamiliar place is an indispensable operational criterion of the paradigm. The lack of the moderate stress, which would have been necessary for the activation of the attachment behaviour, is indicated by the lack of stress behaviours in dogs in the Hansen Wheat study.

In addition, so far, studies using the SST paradigm have compared the subjects' behaviour towards an unfamiliar person (Stranger), and a *dedicated* caregiver. This individual was the infants' mother/father, and in the case of dogs/wolves the hand‐raiser/owner of the subject. In the Hansen Wheat study, however, the very same “familiar person”, one of the nine caregivers who took care of the animals in their shared enclosure, acted as the potential attachment figure for the subjects. Using the same “familiar person” who had to take care of all 15 subjects (all dogs and wolves) as a potential attachment figure in the SST for all canids, indicates a more complex problem than just a deviation of the essential requirements of the test. The applied socialisation regime could have an effect on the bonding process in dogs because they had to share many caregivers without having a dedicated caregiver of their own, which did not model a human‐companion dog relationship. Similarly, running the SST in an orphanage, with one caregiver for many infants, could yield intriguing results; however, it would not provide valid data about the typical attachment behaviour of a human child. In contrast to wolves, where joint care of offspring is more frequent, in the dog there is no communal puppy rearing. In the Topál et al. (2005) comparative study, each hand‐raiser established an individualised relationship with their companion (dogs and wolves).

Generally, it is surprising that the behaviour patterns of both species (especially dogs) were strikingly different from what had been reported in other studies (e.g., much less play, more exploration), which could be caused by the lack of typical dog‐owner interactions during the socialisation or the specific breed (Alaskan huskies) tested. Some aspects of dogs' rearing conditions “Puppies were not disciplined or trained”. were unconventional or at least unusual in a typical setting in the case of 23‐week‐old family dogs, as most dogs receive some sort of training by the time they are 5 months old. Moreover, all 12 dogs came from only two litters of the same, very special “breed” (Alaskan husky), thus they can neither be regarded as independent samples nor representative of dogs in general. In this case a less homogenic dog group would have provided more generalizable results. (In the Topál et al., 2005 study all 11 hand‐raised dogs were mongrels and also 11 pet dogs of various breeds were tested.)

Unlike dogs, wolves exhibited fear towards strangers (crouching, tail‐tucking), which may indicate a suboptimal socialisation regime, as no such behaviours were reported by similar studies on hand‐reared wolves (especially in a familiar environment). This could, at least partly, explain most of their differentiation between the familiar and unfamiliar person (preference for the familiar caregiver). Therefore, opposed to the authors' interpretation, wolves did not show the typical behaviour patterns of attachment, but discriminated between the two persons, which is a necessary but not sufficient condition of attachment (and may have resulted from not being sufficiently socialised to humans). To fulfil the criteria of attachment, wolves should have shown systematic differences in many aspects, which they did not. In fact, in this study even dogs did not behave according to the criteria of attachment, due to the problematic experimental procedure (familiar location, lack of the individualised bond with one specific caregiver). In contrast, in the Topál et al. (2005) “Dog puppies of different socialization history selectively responded to the separation from the owner (stood by the door significantly more upon separation, tended to follow the owner leaving the enclosure, played significantly more with the owner and obtained significantly higher scores upon greeting the owner)”. In the Hansen Wheat et al. study, the wolves did not respond according to the criteria in several relevant situations. For example, unlike dogs, wolves showed a high tendency to follow the leaving stranger (tried to leave the testing location) even in the presence of the owner. This finding is in sharp contrast to the idea that the human caregiver serves as a secure base for wolves.

The authors argue that wolves' “stress response (pacing) was buffered by the presence of a familiar person”. However, this “buffer” effect (in dogs see Gácsi et al., 2013) could be claimed only if the wolves had paced less in the presence of the stranger when the caregiver was also present, that is, in Episode 2 (safe haven effect), compared to the next episode when the subjects were alone with the stranger (unlike wolves, dogs showed exactly this tendency, even though they were not much stressed in the familiar room).

Note, however, that young hand raised and intensely socialised wolves inevitably develop an affiliative bond with their caregiver, and show preference towards them in many respects (e.g., Gácsi et al., 2005). We have never argued that the attachment behaviour pattern present in family dogs emerged out of thin air during domestication. Although both captive wolves and family dogs depend on humans during their whole lives (but dogs' behaviour shows more dependence; see Range & Marshall‐Pescini, 2022), only in case of adult dogs do we have solid data on life‐long attachment (for a review see Topál & Gácsi, 2012), as comparative experiments in wolves are lacking or did not provide such a uniform picture, partly because of the diverging methods used.

Dogs provide further evidence for possessing a functional attachment system. Earlier research from multiple laboratories, using the same methodology (SST) and large number of adult dogs has established that the attachment system of dogs and humans share most significant features; the owner's role as a secure base (Palmer & Custance, 2008) and as safe haven (Gácsi et al., 2013), and that dogs even develop new attachment relationship in adulthood (shelter dogs: Carreiro et al., 2022; Gácsi et al., 2001, and guide dogs for the blind: Valsecchi et al., 2010). Dogs' attachment scores, calculated from the SST, were reported to be associated with their brain activity during sleep at an unfamiliar place with the owner (Carreiro et al., 2022) and with their neural reward responses to verbal praise in an fMRI study (Gábor et al., 2021). Thus, we can conclude that, unless more systematic empirical research on wolves indicates otherwise, the dog‐human bond has specific characteristics that have not been observed so far in wolf pups, and especially not in adult wolves.

Comparative studies often suffer from a problem of preconception. Depending on the authors' views they either claim species differences (divergence) or rather species similarities, including common evolutionary origin (homology) (Kubinyi et al., 2022). We do not claim that attachment is an all‐or‐nothing phenomenon or that we can completely rule out that wolf pups' relationship with the caregiver may fulfil all four operational criteria for attachment, but there is currently no convincing evidence to support this. We cannot refer to any bond as an “attachment‐like” relationship, just because attachment and most social preferences are governed by partially overlapping behaviour controlling mechanisms. In the case of “attachment” and the SST, adapting the human terminology and methodology seems optimal to refer to a functional analogy between the human and dog social systems.

In sum, Hansen Wheat et al. did not present persuading evidence that “wolves can show attachment behaviours towards humans comparable to those of dogs”. When dealing with a behaviour system (Sroufe & Waters, 1977), the subjects' behaviour must meet consistent criteria (Bowlby, 1969; Rajecki et al., 1978), and a few behavioural preferences should not be used to claim the presence of an attachment system, especially, if the experiment violates basic assumptions of the original test. We believe, the intriguing scientific question is whether the dog‐owner relationship is qualitatively different from what could be observed in the wolf‐hand raiser relation. Assessing all available data, our answer is still yes; dogs are unique in this respect.

## AUTHOR CONTRIBUTIONS

**Márta Gácsi:** Conceptualization (equal); writing – original draft (equal). **Ádám Miklósi:** Conceptualization (equal); writing – review and editing (equal). **József Topál:** Conceptualization (equal); writing – review and editing (equal).

## Data Availability

No new data was generated for this manuscript.
